# Shared Behavioural Determinants of Short Sleep and Obesity From Childhood to Adolescence: An Analyses of Growing Up in Scotland Birth Cohort 1

**DOI:** 10.1111/ijpo.70142

**Published:** 2026-08-05

**Authors:** Emma Louise Gale, Joanne Elizabeth Cecil, Andrew James Williams

**Affiliations:** ^1^ School of Medicine University of St Andrews Scotland UK; ^2^ Scottish Collaboration for Public Health Research and Policy, School of Health in Social Sciences University of Edinburgh Edinburgh UK

**Keywords:** adolescent, BMIp, health trajectories, paediatric

## Abstract

**Background:**

Previous research shows a longitudinal relationship between poor sleep and obesity in adolescents, highlighting the need to identify shared modifiable determinants to strengthen health‐promoting interventions. The aim of this study was to investigate the determinants at 10 and 12 years associated with short sleep and body mass index percentile (BMIp) at 14 years, using data from the Growing Up in Scotland (GUS) study.

**Methods:**

Secondary analyses were conducted using data from GUS Birth Cohort 1, sweeps 8–10 (10–14 years). Sleep duration, emotional wellbeing, physical activity, diet, screentime and risk‐taking behaviours were main‐carer or child‐reported and BMIp was objectively measured. Block‐wise linear regression analyses were used to examine associations with sleep duration and BMIp at age 14 years. Meaningful associations were defined as a ±10% difference (standardised *β* ≥ 0.10 or ≤ −0.10).

**Results:**

Data from 4157 participants (50.2% male) were analysed. In adjusted models, having tried smoking and having tried alcohol at 12 years and being allowed to eat whenever the child wanted at 10 years were meaningfully associated with shorter sleep duration and higher BMIp at 14 years. Maternal BMI was meaningfully associated with BMIp only, while poorer emotional wellbeing and sugar‐sweetened beverage consumption were meaningfully associated with shorter sleep only.

**Conclusions:**

Smoking and alcohol experimentation and less structured eating timing were identified as shared determinants of shorter sleep and higher BMIp in adolescence. These findings suggest that risk‐taking behaviours and eating routines may be important targets for future research and health‐promoting interventions addressing sleep and weight‐related outcomes.

## 1 Introduction

Sleep and obesity are bidirectionally associated through both physiological and behavioural mechanisms [1]. Poor sleep disrupts hormonal regulation, including reductions in leptin and increases in ghrelin [2, 3], which influence appetite and energy balance [4]. Elevated cortisol levels associated with insufficient sleep may contribute to stress‐related metabolic changes and fat accumulation, further increasing the risk of weight gain [5, 6]. Conversely, obesity and related metabolic dysfunctions can impair sleep quality and duration through mechanisms such as sleep apnoea [7], inflammation [8] and disruptions in circadian rhythms [9]. Systematic evidence also highlights the role of pre‐sleep behaviours, including poor sleep hygiene, inconsistent bedtime routines, a later chronotype, delayed sleep onset, and social jetlag, as factors associated with obesity in adolescents [10]. Despite these established associations, current obesity interventions often prioritise dietary and physical activity modification [11, 12, 13], with sleep remaining relatively underused as an intervention target [14, 15, 16].

Adolescence is a period marked by rapid physiological, behavioural, and social change, during which both sleep patterns and body weight undergo significant transitions [17, 18, 19]. In adolescents experiencing pubertal hormonal shifts, these sleep and weight‐related changes occur alongside major shifts in behaviour, including increasing mental health concerns [20], independence over eating patterns [21, 22], screen use [23, 24], physical activity [25, 26], social routines and risk‐taking behaviours [27, 28].

Identifying shared behavioural determinants of poor sleep and obesity is essential for developing integrated interventions [29]. A recent systematic review highlighted socio‐environmental determinants (e.g., gender, ethnicity, pubertal status, academic attainment), behavioural factors (e.g., timing of physical activity, unhealthy dietary choices and their timing, screen time, and video gaming quantity and timing) and health determinants (e.g., wellbeing) associated with sleep and obesity in adolescents aged 8–18 years [29]. These findings suggest that sleep and obesity may be influenced not only by the quantity of health behaviours but also by their timing, context and clustering during adolescence. This is particularly relevant because adolescence is characterised by increasing autonomy, changing peer influence and less caregiver‐directed routines, which may shape eating timing, screen use, physical activity and engagement in health‐risk behaviours [18, 19]. While risk‐taking behaviours, such as smoking and alcohol experimentation, were not identified as a primary determinant domain in the systematic review, they may be relevant to examine because they are associated with self‐regulation, decision‐making and broader patterns of health behaviour [28, 30, 31]. Similarly, digital access and online activity behaviours may be relevant because screen‐related effects on sleep and weight status may depend on timing, location, content and caregiver monitoring, rather than total screen time quantity alone [32]. Examining these behavioural domains together may therefore help identify whether specific behaviours act as shared determinants of both sleep duration and body mass index percentile ([20]) during adolescence.

Emotional wellbeing may also play an important role in these relationships. Poorer emotional wellbeing has been associated with shorter sleep duration, disrupted sleep patterns and weight‐related outcomes, potentially through stress‐related physiological pathways and behavioural mechanisms such as emotional eating, reduced physical activity and less consistent routines. Previous analyses of the Growing Up in Scotland (GUS) cohort have shown that sleep duration, emotional wellbeing and BMIp follow interrelated developmental patterns, as well as bidirectional associations between childhood and adolescence, with declines in sleep and emotional wellbeing coinciding with increases in obesity prevalence during early adolescence [33, 34].

Longitudinal cohort studies are important for examining whether earlier behavioural factors are associated with later sleep and weight‐related outcomes. Previous analyses of the GUS cohort have shown that sleep duration declines below recommended levels between ages 8 and 14 [33], while other research has reported marked increases in obesity prevalence between ages 11 and 14 [35]. This developmental period may therefore represent an important window for identifying modifiable behaviours before poor sleep and higher weight status become established [36]. The aim of this study was to examine shared behavioural determinants at 10 and 12 years associated with sleep duration and BMIp at 14 years using longitudinal data from the GUS study. By examining behavioural domains identified as relevant to sleep and obesity, alongside additional age‐relevant behaviours such as risk‐taking and digital activity, this study sought to identify potential targets for integrated interventions to improve adolescent health outcomes.

## 2 Methods and Materials

### 2.1 Ethical Approval

Ethical approval for this study was granted by the School of Medicine Ethics Committee, acting on behalf of the University Teaching and Research Ethics Committee (UTREC) at the University of St Andrews (Approval Code: MD16516, approved 29 September 2022). The secondary dataset used (Growing Up in Scotland, SN 5760) was accessed via the UK Data Service under a Special Licence (Project 226 100). The data were de‐identified and anonymised prior to us receiving them.

### 2.2 Study Design, Recruitment and Data Collection

The Growing Up in Scotland (GUS) study is a longitudinal research initiative that examines children's health, behaviour and development from infancy (first sweep at 10 months old) [37] through early adolescence (sweep 10 at 14 years old) [38]. The first sweep of data was collected in 2005/06 when children were 10 months old, and the tenth sweep was collected in 2018/19 at 14 years old. Participants, along with their primary caregivers, were randomly selected from Child Benefit records provided by HM Revenue and Customs and invited to participate [39]. The initial cohort comprised 5271 children and their caregivers in Scotland [39]. To bolster the sample size, 502 additional participants were included in 2018 [40]. By sweep nine, the retention rate was 56%, dropping slightly to 51% by sweep ten, which included the additional recruits [40]. Ethical approval for sweeps 1–8 was granted by the Scotland ‘A’ Medical Research Ethics Committee (MREC) (application reference 04/MRE10/59), and sweeps 9 and 10 by the NatCen Research Ethics Committee [41]. The GUS study included children who were: (i) born between June 2004 and May 2005, and (ii) living within a Scottish local authority. Boost sample eligibility criteria included: (i) mothers aged 16–24 years at the time of the child's birth and/or (ii) residence in the 15% most deprived areas as identified by the 2016 Scottish Index of Multiple Deprivation (SIMD).

For the current secondary analysis, data were extracted from sweeps 8, 9 and 10, when children were approximately 10, 12 and 14 years old, respectively. These sweeps included objective measurements and questionnaire or interview data from children and their main carers, covering health, wellbeing, diet, physical activity, behaviour, school and home life. Sweeps 8 and 9 were conducted through in‐home interviews by trained interviewers [42, 43], while sweep 10 used a mixed approach, with face‐to‐face interviews conducted between January and March 2020 before data collection moved to telephone or online methods from March to December 2020 due to the COVID‐19 pandemic [40].

### 2.3 Measures

#### 2.3.1 Demographics

##### 2.3.1.1 Child‐Specific Demographics

In each sweep the main carer reported the child's age and sex [38, 44, 45]. In sweep 10, children reported their puberty status through gender‐specific questions [38]. Male‐specific questions included: ‘Have you noticed a deepening in your voice?’ and ‘Has hair begun to grow on your face?’ if the child answered yes to either of those questions, they were then asked at what age they noticed the change [38]. Female‐specific questions included ‘have you ever menstruated (had your period)?’ if the child answered yes, they were then asked at what age the first menstruation occurred [38].

##### 2.3.1.2 Caregiver‐Specific Demographics

The main carer reported the ethnicity and education level of themselves and their partner. Socioeconomic status (SES) across all sweeps was assessed using the National Statistics Socio‐Economic Classification (NS‐SEC), based on the occupation and employment status of the main caregiver or their partner [46].

#### 2.3.2 Anthropometrics

In sweeps 8–10, the child's Body Mass Index percentile (BMIp) was determined using the formula weight (kg) divided by height squared (m^2^), with height and weight measurements taken by trained professionals [38, 44, 45], and age‐ and sex‐standardised percentile estimates were derived using the UK90 reference growth curves [47].

#### 2.3.3 Sleep

In sweep 10, the child participant self‐reported their sleep duration (hours) on a typical night (Table S1) [38].

#### 2.3.4 Emotional Wellbeing

Emotional wellbeing of the child participant was assessed using the emotional symptom subscale from The Strengths and Difficulties Questionnaire (SDQ) [48], recorded in sweep 8 by the main carer [44] and sweep 10 by the child [38]. A higher score indicated poorer emotional wellbeing.

#### 2.3.5 Physical Activity

During sweeps 8 and 9, the main caregiver provided information on the child's physical activity, including the number of hours spent doing physical activity per week and whether the child played team or individual sports weekly (yes/no) (Table S1) [44, 45].

#### 2.3.6 Diet and Consumption Behaviours

At sweep 8, main caregivers responded to a series of questions regarding the child's dietary choices and meal habits. These included the frequency of consuming sweets, chocolates, crisps, sugar‐sweetened beverages (SSBs), fruits and vegetables, as well as how often the child eats breakfast, eats freely or on their own schedule and uses electronic devices during meals (Table S1) [44].

#### 2.3.7 Screen Time

In sweeps 8 and 9, both the main caregiver and the child provided information on screen time habits, including its duration (hours), location, and the caregiver's awareness of the child's screen usage (Table S1) [44, 45]. At sweep 8, caregivers reported on the quantity of screen time (in hours), such as television and electronic device use on weekdays and weekends, as well as the presence of screen‐related devices in the child's bedroom, including televisions, computers, gaming consoles, internet access and mobile phones (yes/no) (Table S1) [44]. At sweep 9, children reported on their caregiver's awareness of their online activities and whether they had ever misled their caregiver about their screen usage (yes/no) (Table S1) [45].

#### 2.3.8 Risk‐Taking Behaviours

In sweeps 8 and 9, both the main caregiver and the child participant answered questions about the child's risk‐taking behaviours (Table S1) [44, 45]. In sweep 8, the Conduct Problems Subscale of the SDQ [48] was used to assess conduct behaviours reported by the main caregiver (Table S1) [44]. A higher score indicating more conduct problems. In sweep 9, the child participant provided self‐reported data on law‐breaking behaviours, such as shoplifting, stealing money, carrying a knife, damaging property, breaking into a property, and graffitiing (yes/no). Additionally, the child reported on their smoking history and alcohol consumption (yes/no) (Table S1) [45].

### 2.4 Data Analyses

Data were imported and analysed using the Statistical Package for Social Sciences (SPSS 28 Statistics). Missing data were assessed using the Little's Missing Completely at Random (MCAR) test, which statistically estimates the likelihood that missing values within a dataset are missing completely at random [49]. Frequency diagrams and descriptive tables were used to demonstrate sample characteristics. Prior to regression modelling, scatterplots of the continuous variables were visually inspected to assess the assumption of linearity. No evidence of marked non‐linear relationships was observed; therefore, linear regression models were considered appropriate. Pearson correlation tables (continuous variables) and descriptive statistics were used to assess the unadjusted longitudinal relationships between potential determinants and sleep and obesity and to determine variables for adjustment in the linear regression analyses. Block‐wise regression analyses were used to assess the adjusted longitudinal relationships between potential determinants and sleep duration (school night) and BMIp. Block one adjusted for child sex, child emotional wellbeing (at 10 years), SES (reference group: managerial and professional occupations), maternal BMI. Block two adjusted for physical activity (hours per day), screentime (hours watching TV per weekday and per weekend day, and electronic usage per weekday and per weekend day) (at 10 years), tried alcohol (at 12 years), tried smoking (at 12 years), breakfast consumption (at 10 years), frequency of sweets and chocolate, crisps and SSBs consumption (at 10 years). Reference groups used were the categories most frequently reported. For the regression analyses, consumption behaviour variables were collapsed due to small numbers of responses in some categories. Sweet and chocolate, crisp and sugar‐sweetened beverage consumption were recoded from eight frequency categories into three groups: less than weekly consumption, which was used as the reference category; consumption 1–4 times per week; and consumption five or more times per week. Breakfast consumption was analysed using the original four‐category response variable: never, occasionally, quite often and mostly. The two additional eating behaviour variables, how often the child was allowed to eat whatever they wanted and how often the child was allowed to eat whenever they wanted, were dichotomised as always/often versus sometimes/never. Meaningful associations from the regression analyses were identified using a ±10% difference (*β* ≤ −0.1 or ≥ 0.1). This threshold was selected to aid interpretation beyond statistical significance alone and was informed by previous BMIp research, where ≥ 10% differences have been considered clinically meaningful in relation to growth trajectories and weight status [50], and was subsequently adopted for the sleep duration measure to allow consistent interpretation across outcomes.

## 3 Results

### 3.1 Missing Data

This Little's MCAR test (*χ* ^2^ = 1786.975, df = 1401, *p* = 0.001) indicated that missing data were not missing at random [49]. Depending on the statistical test, missing values were handled using listwise deletion for descriptive analyses and frequencies or pairwise deletion for Pearson correlations and regression analyses. Findings were interpreted based on the available sample.

### 3.2 Sample Characteristics

The sample included 4157 participants (50.2% male). Full demographic details are presented in Table 1.

**TABLE 1:** Sample characteristics.

		N	%	Mean	SD
Sex	Male	2085	50.2		
	Female	2072	49.8		
Ethnicity	White	3044	96.4		
	Other ethnic background	113	3.6		
Maternal BMI	At recruitment	3081		26.9	5.5
Maternal education level	Under 16 years	84	15.4		
	16–18 years	363	66.5		
	19–24 years	82	15.0		
	25 years or older	14	2.6		
	Still in full‐time education	3	0.5		
NS‐SEC	Managerial and professional occupations	1818	57.6		
	Intermediate occupations	453	14.3		
	Small employers/self‐employed	221	7.0		
	Lower supervisory and technical occupations	240	7.6		
	Semi‐routine and routine occupations	394	12.5		
	Never worked	31	1.0		
BMIp	14 years	2056		0.6	0.3
Sleep duration (hours)	14 years	2351		7.9	1.1
Emotional wellbeing (SDQ) (/10)	10 years	2589		1.6	1.8
	12 years	2598		1.8	2.1
	14 years	2342		1.8	2.1

Abbreviations: BMI, body mass index; N, number; NS‐SEC, National Statistics Socio‐economic Classification; SD, standard deviation; SDQ, Strengths and Difficulties Questionnaire.

The mean number of hours of physical activity per week was 4.1 ± 3.1 h at 10 years. Regular participation in team sports and individual sports at 12 years was present in 52.8% and 47.1% of participants, respectively (Table 2).

**TABLE 2:** Physical activity variables at 10 and 12 years.

Physical activity variable	Age (years)		N	%	Mean	SD
Physical activity (hours per week)	10		3148		4.1	3.1
Regular team sports	12	Yes	1804	52.8		
		No	1613	47.2		
Regular individual sports	12	Yes	1610	47.1		
		No	1807	52.9		

Abbreviations: N, number; SD, standard deviation.

On average, participants watched TV 6.5 ± 1.5 days per week (interquartile range (IQR) = 0 days) and used electronics 6.0 ± 1.9 days per week (IQR = 1 day) at 10 years (Table 3). Mean number of hours spent using electronics at 10 years on a weekday was 1.5 ± 1.2 h (IQR = 1 h) and on a weekend was 4.3 ± 4.2 h (IQR = 4 h) (Table 3). Other screentime variables, including availability of technology in the child's bedroom and caregiver's knowledge of the child's online activity can be seen in Table 3.

**TABLE 3:** Screen time variable descriptives at 10 and 12 years.

Screen time variables	Age (years)		*N*	%	Mean	SD	Q1	Q3
Watching TV (days per week)	10		3146		6.5	1.5	7	7
Watching TV WD (hours)	10		3099		1.5	1.2	1	2
Watching TV WE (hours)	10		3096		4.6	3.6	2	6
Electronics (days per week)	10		3146		6.0	1.9	6	7
Electronics WD (hours)	10		3048		1.5	1.2	1	2
Electronics WE (hours)	10		3046		4.3	4.2	2	6
TV in the bedroom	10	Yes	1920	61.0				
		No	1228	39.0				
Computer, laptop or tablet in the bedroom	10	Yes	1513	48.1				
		No	1635	51.9				
Games console in the bedroom	10	Yes	1516	48.2				
		No	1632	51.8				
Internet access in the bedroom	10	Yes	1645	52.3				
		No	1503	47.7				
Mobile phone in the bedroom	10	Yes	989	31.4				
		No	2159	68.6				
Caregivers have knowledge of the child's online activity	12	Almost everything	1292	41.8				
		Quite a lot	1205	39.0				
		Just a little	472	15.3				
		Almost nothing	123	4.0				
Caregivers don't know what the child does online	12	Yes	273	8.8				
		No	2821	91.2				
Child lies to caregivers about what they do online	12	Yes	242	7.8				
		No	2850	92.2				

Abbreviations: N, number; Q, quartile; SD, standard deviation; TV, television; WD, weekday; WE, weekend.

Commonly reported responses included that they ‘always’ consumed breakfast (84.2%), ‘never’ used electronics at mealtimes (40.5%), ‘sometimes’ eat whatever they want (62.4%) and the child ‘sometimes’ snacks and eats whenever they want (55.1%) (Table 4). Most participants reported their sweets and chocolate consumption as ‘once a day’ (37.4%), crisps consumption ‘2–4 times a week’ (31.8%) and ‘never’ for consuming SSBs (23.5%). Mean pieces of fruit consumed were 1.7 ± 1.4 per day and mean pieces of vegetables consumed were 1.5 ± 1.4 per day.

**TABLE 4:** Diet choice and consumption behaviour variable descriptives at 10 years.

Diet variables	Age (years)		N	%	Mean	SD
Breakfast consumption	10	Never	53	1.7		
		Occasionally	218	6.9		
		Quite often	226	7.2		
		Always	2651	84.2		
Electronics during mealtimes	10	Always	276	8.8		
		Often	434	13.8		
		Sometimes	1160	36.9		
		Never	1273	40.5		
Child eats whatever they want	10	Always	67	3.0		
		Often	250	9.9		
		Sometimes	1735	62.4		
		Never	1094	24.6		
Child snacks and eats whenever they want	10	Always	67	2.1		
		Often	250	7.9		
		Sometimes	1735	55.1		
		Never	1094	34.8		
Sweets and chocolate consumption	10	More than once a day	321	10.2		
		Once a day	1176	37.4		
		4–6 times a week	365	11.6		
		2–4 times a week	908	28.8		
		Once a week	270	8.6		
		1–3 times a month	65	2.1		
		Less than once a month	25	0.8		
		Never	18	0.6		
Crisps consumption	10	More than once a day	103	3.3		
		Once a day	914	29.0		
		4–6 times a week	381	12.1		
		2–4 times a week	1002	31.8		
		Once a week	422	13.4		
		1–3 times a month	160	5.1		
		Less than once a month	77	2.4		
		Never	89	2.8		
SSB consumption	10	More than once a day	549	17.4		
		Once a day	427	13.6		
		4–6 times a week	115	3.7		
		2–4 times a week	326	10.4		
		Once a week	381	12.1		
		1–3 times a month	300	9.5		
		Less than once a month	309	9.8		
		Never	741	23.5		
Fruits (per day)	10		3135		1.7	1.4
Vegetables (per day)	10		3133		1.5	1.4

Abbreviations: N, number; SD, standard deviation; SSB, sugar‐sweetened beverage.

Mean conduct problem score at 10 and 12 years was 1.2 ± 1.4 and 1.2 ± 1.7, respectively. Other risk‐taking behaviours at 10 and 12 years are recorded in Table 5.

**TABLE 5:** Risk‐taking behaviour variable descriptives at 10 and 12 years.

Risk‐taking behaviour variables	Age (years)		N	%	Mean	SD
Conduct problems	10		3134		1.2	1.4
	12		3338		1.2	1.7
Tried a cigarette	12	Yes	128	3.9		
		No	3156	96.1		
Tried vaping	12	Yes	230	7.0		
		No	3054	93.0		
Tried alcohol	12	Yes	671	20.4		
		No	2613	79.6		
Age of first alcoholic drink	12	Age 8 or under	42	6.3		
		Age 9 or 10	137	20.4		
		Age 11 and over	491	73.3		
Drank alcohol in the last 30 days	12	Never	527	78.5		
		1–2 days	112	16.7		
		3–5 days	13	1.9		
		6–9 days	4	0.6		
		10–19 days	4	0.6		
		20–30 days	11	1.6		
Stolen from a shop	12	Yes	207	6.3		
		No	3078	93.7		
Stolen money	12	Yes	343	10.4		
		No	2942	89.6		
Carried a weapon	12	Yes	50	1.5		
		No	3235	98.5		
Damaged a property	12	Yes	96	2.9		
		No	3189	97.1		

Abbreviations: N, number; SD, sleep duration.

### 3.3 Determinants of Higher Body Mass Index Percentile

Overall model 1 (*R* ^2^ = 0.072, adjusted *R* ^2^ = 0.067, *F* = 13.817, *p* < 0.001; Table 6a) and model 2 were significant (*R* ^2^ = 0.183, adjusted *R* ^2^ = 0.169, Δadjusted *R* ^2^ = 0.102, *F* = 16.000, *p* < 0.001) (Table 6a).

**TABLE 6a:** Model summary of the regression analyses of the determinants (at 10 and 12 years) of body mass index percentile (at 14 years).

Model	*R* ^2^	Adj *R* ^2^	∆Adj *R* ^2^	*F*	*p*
1	0.072	0.067		13.817	< 0.001
2	0.183	0.169	0.102	16.000	< 0.001

*Note:* Dependent variable: Child body mass index percentile. Model 1: Adjusted for Child gender, maternal BMI, child emotional wellbeing, NSSEC group (reference group: managerial and professional occupations). Model 2: Adjusted for model 1 + physical activity (hours per week) (10 years), time spent watching TV on a weekday (hours) (10 years), time spent watching TV on a weekend day (hours) (10 years), time spent using electronics on a weekday (hours) (10 years), time spent using electronics on a weekend day (hours) (10 years), tried smoking (12 years), tried alcohol (12 years), breakfast consumption, eating whenever the child wants, sweets and chocolate consumption (reference group: less than weekly) (10 years), crisps consumption (reference group: less than weekly) (10 years), SSB consumption (reference group: less than weekly) (10 years).

Using model 2, maternal BMI was meaningfully associated with higher BMIp at 14 years (standardised *β* = 0.241, *t* = 9.634, *p* < 0.001, 95% CI = 0.010 to 0.296). Having tried smoking at 12 years (standardised *β* = 0.103, *t* = 2.989, *p* = 0.026, 95% CI = 0.012 to 0.167), having tried alcohol at 12 years (standardised *β* = 0.133, *t* = 3.471, *p* = 0.014, 95% CI = 0.010 to 0.185), and being allowed to eat whenever the child wanted at 10 years (standardised *β* = 0.101, *t* = 2.628, *p* = 0.009, 95% CI = 0.019 to 0.282) were also meaningfully associated with higher BMIp at 14 years (Table 6b).

**TABLE 6b:** Regression analyses of determinants (at 10 and 12 years) of body mass index percentile (at 14 years).

Model		Unst. β	SE	Stand. β	*t*	*p*	CI 95%	
							Lower	Upper
1	(Constant)	0.245	0.038		6.510	< 0.001	0.171	0.319
	Child gender	0.013	0.014	0.022	0.912	0.362	−0.015	0.041
	Maternal BMI	0.013	0.001	**0.242**	9.762	< 0.001	0.010	0.296
	Child emotional wellbeing (10 years)	0.009	0.004	**0.104**	2.186	0.029	0.001	0.117
	NS‐SEC (Ref group: managerial and professional occupations)							
	*Intermediate occupations*	0.031	0.020	0.041	1.577	0.115	−0.008	0.069
	*Small employers/self‐employed*	0.024	0.027	0.023	0.894	0.371	−0.029	0.077
	*Lower supervisory and technical occupations*	0.033	0.036	0.023	0.924	0.355	−0.038	0.105
	*Semi‐routine and routine occupations*	0.015	0.019	0.021	0.793	0.428	−0.022	0.052
	*Not working*	−0.015	0.078	−0.005	−0.186	0.853	−0.168	0.139
2	(Constant)	0.274	0.065		4.198	< 0.001	0.246	0.501
	Child gender	0.012	0.014	0.020	0.826	0.409	−0.016	0.040
	Maternal BMI	0.013	0.001	**0.241**	9.634	< 0.001	0.010	0.296
	Child emotional wellbeing (10 years)	0.008	0.004	0.047	2.868	0.042	0.008	0.066
	NS‐SEC (Ref group: managerial and professional occupations)							
	*Intermediate occupations*	0.027	0.02	0.036	1.369	0.171	−0.012	0.066
	*Small employers/self‐employed*	0.019	0.027	0.018	0.714	0.476	−0.034	0.072
	*Lower supervisory and technical occupations*	0.025	0.037	0.017	0.674	0.500	−0.047	0.096
	*Semi‐routine and routine occupations*	0.012	0.020	0.017	0.614	0.539	−0.026	0.050
	*Not working*	−0.007	0.079	−0.002	−0.091	0.928	−0.162	0.148
	Physical activity (hours per week) (10 years)	0.002	0.003	0.005	0.949	0.343	−0.003	0.008
	Watching TV (hours per weekday) (10 years)	−0.003	0.007	−0.010	−0.387	0.699	−0.016	0.010
	Watching TV (hours per weekend day) (10 years)	0.000	0.002	0.003	0.139	0.889	−0.004	0.004
	Using Electronics (hours per weekday) (10 years)	0.009	0.007	0.037	1.330	0.184	−0.004	0.043
	Using Electronics (hours per weekend day) (10 years)	0.089	0.016	0.073	2.689	0.044	0.004	0.093
	Tried smoking (12 years)	0.117	0.046	**0.103**	2.989	0.026	0.012	0.167
	Tried alcohol (12 years)	0.148	0.019	**0.133**	3.471	0.014	0.010	0.185
	Breakfast consumption (10 years)	−0.035	0.030	−0.030	−1.173	0.241	−0.094	0.024
	Child eats whenever (10 years)	0.158	0.026	**0.101**	2.628	0.009	0.019	0.282
	Sweet and chocolate consumption (Ref: < weekly) (10 years)							
	*1–4 times a week*	−0.017	0.042	−0.029	−0.404	0.686	−0.100	0.066
	*5+ times a week*	−0.021	0.042	−0.035	−0.492	0.623	−0.103	0.062
	Crisps consumption (Ref group: < weekly) (10 years)							
	*1–4 times a week*	−0.006	0.025	−0.011	−0.248	0.804	−0.055	0.043
	*5+ times a week*	−0.002	0.026	−0.003	−0.073	0.941	−0.052	0.048
	SSB consumption (Ref group: < weekly) (10 years)							
	*1–4 times a week*	0.024	0.018	0.035	1.319	0.187	−0.012	0.059
	*5+ times a week*	−0.008	0.017	−0.013	−0.484	0.629	−0.041	0.025

*Note:* Dependent variable: Child body mass index percentile. Meaningful associations indicated by bold stand. β ≥ 0.10 or ≤ −0.10.

Abbreviations: BMI, body mass index; NS‐SEC, National Statistics Socio‐economic Classification; Ref, reference group; SSB, sugar sweetened beverage.

### 3.4 Determinants of Shorter Sleep Duration

Overall model 1 (*R* ^2^ = 0.038, adjusted *R* ^2^ = 0.027, *F* = 5.026, p < 0.001; Table 7a) and model 2 were significant (*R* ^2^ = 0.189, adjusted *R* ^2^ = 0.151, Δadjusted *R* ^2^ = 0.124, *F* = 10.598, p < 0.001) (Table 7a).

**TABLE 7a:** Model summary of the regression analyses of the determinants (at 10 and 12 years) of sleep duration (school night) (at 14 years).

Model	*R* ^2^	Adj *R* ^2^	∆Adj *R* ^2^	*F*	*p*
1	0.038	0.027		5.026	< 0.001
2	0.189	0.151	0.124	10.598	< 0.001

*Note:* Dependent variable: Child sleep duration (school night). Model 1: Adjusted for child gender, maternal BMI, child emotional wellbeing, NSSEC group (reference group: managerial and professional occupations). Model 2: Adjusted for model 1 + physical activity (hours per week) (10 years), time spent watching TV on a weekday (hours) (10 years), time spent watching TV on a weekend day (hours) (10 years), time spent using electronics on a weekday (hours) (10 years), time spent using electronics on a weekend day (hours) (10 years), tried smoking (12 years), tried alcohol (12 years), breakfast consumption, eating whenever the child wants, sweets and chocolate consumption (reference group: less than weekly) (10 years), crisps consumption (reference group: less than weekly) (10 years), SSB consumption (reference group: less than weekly) (10 years).

Using model 2, poorer child emotional wellbeing at 10 years was meaningfully associated with shorter sleep duration at 14 years (standardised *β* = −0.105, *t* = −3.214, *p* = 0.001, 95% CI = −0.133 to −0.018) (Table 7b). Having tried smoking at 12 years (standardised *β* = −0.233, *t* = −3.682, *p* < 0.001, 95% CI = −0.810 to −0.180), having tried alcohol at 12 years (standardised *β* = −0.143, *t* = −3.009, *p* = 0.008, 95% CI = −0.273 to −0.016), and being allowed to eat whenever the child wanted at 10 years (standardised *β* = −0.182, *t* = −2.946, *p* = 0.004, 95% CI = −0.254 to −0.090) were meaningfully associated with shorter sleep duration at 14 years (Tables 7b and Table 7b). More frequent SSB consumption was meaningfully associated with longer sleep duration at 14 years (standardised *β* = 0.103, *t* = 2.740, *p* = 0.002, 95% CI = 0.014 to 0.231) (Table 7b).

**TABLE 7b:** Regression analyses of determinants (at 10 and 12 years) of sleep duration (school night) (at 14 years).

Model		Unst. β	SE	Stand. β	*t*	*p*	CI 95%	
							Lower	Upper
1	(Constant)	8.296	0.128		64.576	**< 0.001**	8.044	8.548
	Child gender	0.032	0.050	0.015	0.633	0.527	−0.066	0.130
	Maternal BMI	−0.009	0.005	−0.015	−1.922	0.055	−0.018	0.000
	Child emotional wellbeing (10 years)	−0.061	0.014	**−0.101**	−4.318	**< 0.001**	−0.088	−0.033
	NS‐SEC (Ref group: managerial and professional occupations)							
	*Intermediate occupations*	−0.053	0.068	−0.019	−0.773	0.439	−0.186	0.081
	*Small employers/self‐employed*	0.007	0.093	0.002	0.073	0.942	−0.175	0.189
	*Lower supervisory and technical occupations*	0.043	0.125	0.008	0.342	0.732	−0.203	0.289
	*Semi‐routine and routine occupations*	−0.207	0.066	−0.079	−3.145	**0.002**	−0.336	−0.078
	*Not working*	−0.467	0.298	−0.036	−1.564	0.118	−1.052	0.119
2	(Constant)	8.058	0.227		35.467	**< 0.001**	7.612	8.503
	Child gender	0.028	0.049	0.013	0.567	0.571	−0.069	0.125
	Maternal BMI	−0.005	0.005	−0.017	−1.154	0.248	−0.024	0.004
	Child emotional wellbeing (10 years)	−0.045	0.014	**−0.105**	−3.214	**0.001**	−0.133	−0.018
	NS‐SEC (Ref group: managerial and professional occupations)							
	*Intermediate occupations*	0.009	0.068	0.003	0.130	0.896	−0.125	0.143
	*Small employers/self‐employed*	0.039	0.092	0.010	0.426	0.670	−0.141	0.219
	*Lower supervisory and technical occupations*	0.148	0.125	0.028	1.186	0.236	−0.097	0.394
	*Semi‐routine and routine occupations*	−0.089	0.068	−0.034	−1.319	0.187	−0.222	0.044
	*Not working*	−0.222	0.298	−0.017	−0.745	0.456	−0.806	0.362
	Physical activity (hours per week) (10 years)	0.053	0.009	0.089	3.679	**< 0.001**	0.015	0.151
	Watching TV (hours per weekday) (10 years)	−0.034	0.024	−0.035	−1.440	0.150	−0.081	0.012
	Watching TV (hours per weekend day) (10 years)	−0.004	0.008	−0.012	−0.510	0.610	−0.019	0.011
	Using Electronics (hours per weekday) (10 years)	−0.022	0.025	−0.023	−0.902	0.367	−0.070	0.026
	Using Electronics (hours per weekend day) (10 years)	−0.097	0.006	−0.088	−2.133	**0.047**	−0.099	−0.002
	Tried smoking (12 years)	−0.495	0.161	**−0.233**	−3.682	**< 0.001**	−0.810	−0.180
	Tried alcohol (12 years)	−0.144	0.066	**−0.143**	−3.009	**0.008**	−0.273	−0.016
	Breakfast consumption (10 years)	0.152	0.105	0.054	1.446	0.148	−0.034	0.359
	Child eats whenever (10 years)	−0.183	0.088	**−0.182**	−2.946	**0.004**	−0.254	−0.090
	Sweet and chocolate consumption (Ref: < weekly) (10 years)							
	*1–4 times a week*	0.012	0.151	0.005	0.079	0.937	−0.284	0.308
	*5+ times a week*	−0.028	0.150	−0.013	−0.188	0.851	−0.323	0.266
	Crisps consumption (Ref group: < weekly) (10 years)							
	*1–4 times a week*	−0.019	0.086	−0.009	−0.218	0.827	−0.186	0.149
	*5+ times a week*	−0.072	0.088	−0.033	−0.815	0.415	−0.245	0.101
	SSB consumption (Ref group: < weekly) (10 years)							
	*1–4 times a week*	0.109	0.062	0.103	2.740	**0.002**	0.014	0.231
	*5+ times a week*	−0.022	0.058	−0.010	−0.383	0.702	−0.137	0.092

*Note:* Dependent variable: Child sleep duration (school night). Meaningful associations indicated by bold stand. β ≥ 0.10 or ≤ −0.10.

Abbreviations: NS‐SEC, National Statistics Socio‐economic Classification; Ref, reference group; SSB, sugar sweetened beverage.

### 3.5 Shared Determinants of Higher Body Mass Index Percentile and Shorter Sleep Duration

Across the fully adjusted models (Tables 6a, 6b, 7a, and 7b), having tried smoking at 12 years, having tried alcohol at 12 years, and being allowed to eat whenever the child wanted at 10 years were meaningfully associated with both BMIp and sleep duration at 14 years, defined as a standardised *β* of ≥ 0.10 or ≤ −0.10. Maternal BMI was meaningfully associated with BMIp only, and emotional wellbeing at 10 years and SSB consumption 1–4 times per week were meaningfully associated with sleep duration only.

Several variables were statistically significantly associated with the outcomes but did not meet the threshold for a meaningful association. Emotional wellbeing at 10 years was statistically significantly associated with BMIp, but the standardised *β* did not meet the meaningful association threshold (standardised *β* = 0.047, *t* = 2.868, *p* = 0.042, 95% CI = 0.008 to 0.066). Electronic use on a weekend day was statistically significantly associated with both BMIp and sleep duration but did not meet the meaningful association threshold in either model. Higher electronic use on a weekend day was associated with higher BMIp (standardised *β* = 0.073, *t* = 2.689, *p* = 0.044, 95% CI = 0.004 to 0.093) and shorter sleep duration (standardised *β* = −0.088, *t* = −2.133, *p* = 0.047, 95% CI = −0.099 to −0.002). Physical activity was also statistically significantly associated with sleep duration, but the standardised *β* did not meet the meaningful association threshold (standardised *β* = 0.089, *t* = 2.130, *p* < 0.001, 95% CI = 0.015 to 0.151).

## 4 Discussion

The aim of this secondary data analysis was to examine whether behavioural determinants at 10 and 12 years were associated with both sleep duration and BMIp at 14 years in the Growing Up in Scotland cohort. In fully adjusted models, which accounted for socioeconomic inequalities through adjustment for NS‐SEC group alongside demographic, behavioural, and lifestyle factors, having tried smoking at 12 years, having tried alcohol at 12 years, and being allowed to eat whenever the child wanted at 10 years were meaningfully associated with both shorter sleep duration and higher BMIp at 14 years.

Smoking and alcohol experimentation at 12 years were meaningfully associated with both shorter sleep duration and higher BMIp at 14 years, suggesting that early engagement in substance‐related risk‐taking behaviours may indicate a broader pattern of poorer behavioural health. Risk‐taking behaviours often increase during adolescence alongside greater autonomy and changing peer and social contexts [51]. While some risk‐taking may be developmentally normative and contribute to autonomy and adaptive decision‐making [52, 53], behaviours such as smoking and alcohol use are associated with adverse physical and mental health outcomes [54, 55]. Previous research has also reported associations between adolescent risk‐taking behaviours and shorter sleep duration [56, 57, 58], increased sleep fragmentation and disturbance [59, 60, 61, 62], poorer sleep habits [60] and chronic sleep deprivation [59].

Short sleep has previously been proposed as a determinant of risk‐taking behaviour, potentially through impaired decision‐making and self‐regulation [57]. However, emerging evidence suggests that the relationship between risk‐taking behaviours and short sleep may be bidirectional [63, 64]. Smoking and alcohol experimentation may also co‐occur with other behaviours relevant to sleep and weight status, including poorer dietary choices [65], higher energy drink or SSB consumption [66] and greater screen time [67]. In the current analyses, SSB consumption was meaningfully associated with sleep duration, but not BMIp, suggesting that some dietary behaviours may be more strongly associated with sleep than weight status in this cohort. In addition, risk‐taking behaviours have been associated with poorer wellbeing and the development of mental health difficulties [68, 69, 70, 71, 72], suggesting that wellbeing may be an important factor in the relationship between risk‐taking behaviours, sleep and BMIp. The clustering of these behaviours may partly explain why smoking and alcohol experimentation remained associated with both sleep duration and BMIp after adjustment for sociodemographic, wellbeing and behavioural covariates. However, given the observational design, these findings should be interpreted as longitudinal associations and do not indicate causality.

Being allowed to eat whenever the child wanted at 10 years was meaningfully associated with both shorter sleep duration and higher BMIp at 14 years. This variable may reflect lower structure around eating routines, greater autonomy over food timing or more frequent eating outside planned meals. The timing and regularity of food consumption may be particularly relevant during adolescence, when young people gain increasing autonomy over health‐related behaviours, including food choices and eating occasions [73, 74, 75, 76, 77]. Irregular or later food consumption, excessive sugar intake and caffeine consumption have been associated with delayed circadian timing, later bedtimes, sleep fragmentation and shorter sleep duration [78, 79, 80, 81, 82, 83]. Shorter sleep and later bedtimes may also affect decision‐making [57, 84, 85, 86], increase portion size [4] and increase cravings for less healthy foods [87]. These pathways may help explain why less structured eating routines were associated with both shorter sleep duration and higher BMIp in the current analyses.

In contrast, individual dietary frequency variables, including breakfast consumption, sweets and chocolate and crisp consumption, were not meaningfully associated with shorter sleep duration and higher BMIp in the fully adjusted models. More frequent SSB consumption was meaningfully associated with sleep duration but not BMIp. This may reflect limitations in the dietary measure, which captured frequency of consumption but not portion size, total volume, sugar content, caffeine content, timing of intake, total energy intake or overall dietary quality. Consequently, the SSB variable may have captured aspects of behavioural routine or consumption timing more closely associated with sleep duration, while being insufficiently detailed to detect an independent association with BMIp. The absence of a meaningful association with BMIp may also reflect the multifactorial nature of weight status, where the independent contribution of a single dietary behaviour may be too small to detect once broader influences, including maternal BMI, socioeconomic status, physical activity, screen time, and other health behaviours, are considered. As dietary behaviours commonly cluster with other lifestyle behaviours during adolescence [88, 89], future research should examine the timing, quantity and broader context of food and drink consumption, including whether SSBs are consumed later in the day or contain caffeine, to clarify their relationship with sleep and weight status.

Some variables were statistically significant but did not meet the predefined threshold for meaningful association. Electronic use on a weekend day was statistically significantly associated with both shorter sleep duration and higher BMIp, but the standardised beta values were below 0.10. This is consistent with previous research indicating associations between screen time, sleep, sedentary behaviour and obesity in adolescents [32, 90, 91, 92]. However, the smaller effect sizes observed in the fully adjusted models suggest that weekend electronic use may have a weaker independent association with sleep duration and BMIp once other sociodemographic, wellbeing and behavioural factors are considered. This may also reflect limitations in the screen time measure available in GUS, which captured the number of hours spent using electronics, but not the timing, context or content of screen use. The timing of screen exposure may be particularly important for sleep, as evening or pre‐sleep screen use may contribute to delayed sleep onset through light exposure, circadian delay and melatonin suppression [32, 93, 94, 95]. Therefore, duration‐based measures may underestimate the relevance of screen behaviours if they do not distinguish between daytime use and evening or bedtime use. Screen use may also cluster with sedentary behaviour, poorer dietary patterns and other health‐related behaviours, making it difficult to isolate its independent contribution to BMIp [90, 91, 92].

Physical activity was statistically significantly associated with sleep duration but did not meet the threshold for meaningful association with BMIp. Previous evidence indicates that physical activity, particularly moderate‐to‐vigorous physical activity, is associated with healthier sleep and weight‐related outcomes in adolescents [96, 97, 98, 99, 100], potentially through improved metabolic regulation, increased energy expenditure and effects on circadian alignment [101, 102, 103, 104, 105]. The smaller association observed in the current study may partly reflect the measurement of physical activity in GUS, which relied on reported quantity of activity rather than objective measures of intensity, timing, duration or domain. This may have introduced measurement variability, particularly if respondents differed in whether they included school‐based activity, active travel or informal play. Future studies using objective measures, such as actigraphy, would allow more detailed examination of how different components of physical activity are associated with sleep duration and BMIp [106]. Overall, while screen use and physical activity remain relevant adolescent health behaviours, the current analyses do not support framing them as primary shared determinants of both sleep duration and BMIp in this cohort.

This study has several strengths. It used data from the Growing Up in Scotland cohort, enabling longitudinal analysis of behavioural determinants at 10 and 12 years in relation to sleep duration and BMIp at 14 years. The large sample size strengthened the statistical power of the analyses, while the breadth of the dataset enabled adjustment for sociodemographic, wellbeing and behavioural factors within the same regression models. This is important given that adolescent health behaviours often co‐occur, and the revised modelling approach reduced the risk of overinterpreting multiple separate tests. The use of objectively measured height and weight to derive BMIp is an additional strength.

Several limitations should also be considered. Missing data were not missing completely at random, indicating that the analytical sample may have differed from the full GUS cohort (a nationally representative cohort). Although the original cohort was designed to be nationally representative, attrition and selective missingness may have introduced bias, and caution is therefore required when generalising the findings to the wider adolescent population in Scotland. Participant attendance also varied across sweeps, with reduced participation at sweep 10 during the COVID‐19 pandemic, which may have contributed to variation in the number of valid cases across analyses.

A further limitation relates to the measurement of behavioural determinants. Several variables were based on caregiver or child report, which may be affected by recall bias, interpretation differences or social desirability bias, particularly for sensitive risk‐taking behaviours such as smoking and alcohol experimentation. Some measures were also relatively broad. For example, physical activity was reported as quantity of activity and did not capture intensity, timing, duration or context. Similarly, screen time measures captured hours of use but not timing, content or whether use occurred close to bedtime. Dietary variables captured frequency of consumption but not portion size, total energy intake, timing, caffeine content or broader dietary quality. These measurement limitations may have reduced the ability to detect stronger or more specific associations with sleep duration and BMIp.

Finally, sleep duration was self‐reported using a single item, which may have introduced reporting bias and did not capture sleep quality, sleep timing, variability between school nights and weekends, or symptoms of sleep disturbance. Although the longitudinal design strengthens temporal ordering, causal relationships cannot be inferred. Future research would benefit from objective measures of sleep and physical activity, more detailed measures of screen and dietary behaviours, and repeated assessment of risk‐taking behaviours across later adolescence.

## 5 Conclusion

Overall, the findings suggest that smoking experimentation, alcohol experimentation and less structured eating timing in late childhood and early adolescence were shared determinants of shorter sleep duration and higher BMIp at 14 years. In contrast, maternal BMI was associated with BMIp only, while emotional wellbeing and sugar‐sweetened beverage consumption were associated with sleep duration only. These findings support a more focused approach to future adolescent health research and intervention development, with attention to behavioural clustering, family routines and early risk‐taking behaviours as potential targets for improving both sleep and weight‐related outcomes.

## Author Contributions

All listed authors should have contributed to the manuscript substantially and have agreed to the final submitted version. Conceptualisation and methodology: E.L.G., J.E.C., A.J.W. Data curation, visualisation, project administration and original draft preparation: E.L.G. Investigation and formal analysis: E.L.G., A.J.W. Supervision and funding acquisition: J.E.C. and A.J.W. Review and editing: E.L.G., J.E.C., A.J.W.

## Funding

The authors have nothing to report.

## Ethics Statement

Ethical approval for this study was granted by the School of Medicine Ethics Committee, acting on behalf of the University Teaching and Research Ethics Committee (UTREC) at the University of St Andrews (Approval Code: MD16516, approved 29 September 2022). The secondary dataset used (Growing Up in Scotland, SN 5760) was accessed via the UK Data Service under a Special Licence (Project 226 100). All data were anonymised prior to access and use.

## Consent

This study involved secondary analyses of anonymised data from the Growing Up in Scotland cohort. Informed consent, including parent/guardian consent for participants under the age of 16, was obtained by the original data collectors prior to data being made available. The authors had no direct contact with participants and were not involved in the original consent process.

## Conflicts of Interest

The authors declare no conflicts of interest.

## Supporting information


**Supporting Information: 1** Table titles.
**Table S1:** Physical activity, diet and consumption habits, screen time and risk‐taking behaviour outcomes from the Growing Up in Scotland dataset.
**Supporting Information: 2** Table titles.
**Table S1:1** Differences in mean and confidence intervals of BMIp at 12 and 14 years (sweep 9 and 10), sleep duration at 14 years (sweep 10) by participation in team and individual sports at 12 years (sweep 9).
**Table S1:2** Pearson correlation of the unadjusted longitudinal relationship between physical activity at 10 years (sweep 8), sleep duration at 14 years (sweep 10) and obesity at 12 and 14 years (sweeps 9 and 10).
**Table S2:1** Mean and confidence interval differences in BMIp at 12 and 14 years (sweeps 9 and 10), sleep duration at 14 years and screen time behaviours at 10 and 12 years (sweeps 8 and 9).
**Table S2:2** Pearson correlation of the unadjusted longitudinal relationship between screen time and sleep and obesity.
**Table S3:1** Mean and standard deviations of BMIp at 12 and 14 years (sweep 9 and 10) and sleep duration at 14 years (sweep 10) by diet variable responses at 10 years (sweep 8).
**Table S3:2** Pearson correlations of the longitudinal relationship between diet choices at 10 years (sweep 8) and sleep duration at 14 years (sweep 10) and obesity at 12 and 14 years (sweeps 9 and 10).
**Table S4:1** Mean and confidence interval differences in BMIp at 12 and 14 years, sleep duration at 14 years and risk‐taking at 12 and 14 years.
**Table S4:2** The longitudinal relationship between risk‐taking behaviours and sleep and obesity: Pearson correlation.

## Data Availability

The data that support the findings of this study are available from UK Data Service. Restrictions apply to the availability of these data, which were used under licence for this study. Data are available from https://doi.org/10.5255/UKDA‐Series‐200020 with the permission of UK Data Service.
