# Supplementary material for: Shared Behavioural Determinants of Short Sleep and Obesity From Childhood to Adolescence: An Analyses of Growing Up in Scotland Birth Cohort 1
Source: Pediatr Obes. 2026 Aug 5;21(8):e70142. doi: 10.1111/ijpo.70142 (PMC13439959; doi:10.1111/ijpo.70142)
Supplement: Supplementary file 1 [file IJPO-21-e70142-s001.pdf]

Shared behavioural determinants of short sleep and obesity from childhood to adolescence: an analyses of Growing Up in Scotland birth cohort 1

Running title: Shared behavioural determinants of short sleep and obesity from childhood to adolescence

Keywords: paediatric, BMIp, health trajectories, adolescent

Emma Louise Gale[1*], Joanne Elizabeth Cecil[1] and Andrew James Williams[2]

[1] School of Medicine, University of St Andrews, Scotland, UK

[2] School of Health in Social Science, University of Edinburgh, Scotland, UK

***Corresponding Author:** Emma Louise Gale – eg243@st-andrews.ac.uk, +447703332334, (ORCID ID - 0000-0002-4266-6041), Desk 6.0, Room 226, School of Medicine, University of St Andrews, Scotland, UK

Supplementary material 1

Table S1, physical activity, diet and consumption habits, screen time and risk-taking behaviour outcomes from the Growing Up in Scotland dataset

Topic	Question	Responses	Reported by	Sweep (s)
Sleep fragmentation	Experienced “waking up too much or too early”	“Yes” (1)<br>“No” (0)	Child participant	10
Sleep onset latency	“How long does it take you to fall asleep”	“0-15 minutes” (1)<br>“16-30 minutes” (2)<br>“31-45 minutes” (3)<br>“46-60 minutes” (4)<br>“More than 60 minutes” (5)	Child participant	10
Insomnia symptoms	Experienced “insomnia”	“Yes” (1)<br>“No” (0)	Child participant	10
Oversleeping	Experienced “sleeping too much”	“Yes” (1)<br>“No” (0)	Child participant	10
Sleep duration	“How many hours sleep do you get on a school night?”	Open response (hours)	Child participant	10
	“How many hours sleep do you get on a non-school night?”	Open response (hours)	Child participant	10
Physical activity and sports	“On average, how many hours per week does ^ChildName spend doing physical activity?”	Open response (hours)	Main carer	8
	“Does ^ChildName participate in organised team sports?”	“Yes” (1)<br>“No” (0)	Main carer	9
	“Does ^ChildName participate in organised individual sports?”	“Yes” (1)<br>“No” (0)	Main carer	9
Dietary choices	“How often does ^ChildName eat sweets and chocolates?”	“More than once a day” (1)<br>“Once a day” (2)<br>“5-6 times a week” (3)<br>“2 to 4 times a week” (4)<br>“Once a week” (5)<br>“1-3 times a month” (6)<br>“Less than once a month” (7)<br>“Never” (8)	Main carer	8
	“How often does ^ChildName eat crisps?”	“More than once a day” (1)<br>“Once a day” (2)<br>“5-6 times a week” (3)	Main carer	8

		"2 to 4 times a week" (4)<br>"Once a week" (5)<br>"1-3 times a month" (6)<br>"Less than once a month" (7)<br>"Never" (8)		
	"How often does ^ChildName drink soft drinks not including diet or sugar-free?"	"More than once a day" (1)<br>"Once a day" (2)<br>"5-6 times a week" (3)<br>"2 to 4 times a week" (4)<br>"Once a week" (5)<br>"1-3 times a month" (6)<br>"Less than once a month" (7)<br>"Never" (8)	Main carer	8
	"How many portions of fruit did ^ChildName eat yesterday?"	Open response (number of portions)	Main carer	8
	"How many portions of fruit did ^ChildName eat yesterday?"	Open response (number of portions)	Main carer	8
Meal habits	"How often does ^ChildName eat breakfast?"	"Never" (1)<br>"Occasionally" (2)<br>"Quite often" (3)<br>"Mostly" (4)	Main carer	8
	"How often is ^ChildName allowed to eat whatever they wants?"	"Always" (1)<br>"Often" (2)<br>"Sometimes" (3)<br>"Never" (4)	Main carer	8
	"Do you allow ^ChildName to eat whenever they wants to?"	"Always" (1)<br>"Often" (2)<br>"Sometimes" (3)<br>"Never" (4)	Main carer	8
	"How often does ^ChildName watch television, or use an electronic device, whilst eating their main meal?"	"Always" (1)<br>"Often" (2)<br>"Sometimes" (3)<br>"Never" (4)	Main carer	8
Quantity of screen time	"Thinking about the past week, on how many days did ^ChildName watch television, including watching DVDs or videos on a television, for at least 10 minutes at a time?"	Open response (days)	Main carer	8
	"How long on average would ^ChildName watch TV on a weekday?"	Open response (hours)	Main carer	8

	"On a typical weekday how long on average would ^ChildName watch TV?"	Open response (hours)	Main carer	8
	"Thinking about the past week, on how many days did ^ChildName watch television, including watching DVDs or videos on a television, for at least 10 minutes at a time?"	Open response (days)	Main carer	8
	"On a typical weekday how long, on average, would ^ChildName usually use any electronic device for in total?"	Open response (hours)	Main carer	8
	"On a typical weekend how long, on average, would ^ChildName usually use any electronic device for in total?"	Open response (hours)	Main carer	8
Location of devices	"TV in bedroom"	"Yes" (1)<br>"No" (0)	Main carer	8
	"Computer, laptop or tablet in bedroom"	"Yes" (1)<br>"No" (0)	Main carer	8
	"Games console in bedroom"	"Yes" (1)<br>"No" (0)	Main carer	8
	"Internet access in bedroom"	"Yes" (1)<br>"No" (0)	Main carer	8
	"Mobile phone in bedroom"	"Yes" (1)<br>"No" (0)	Main carer	8
Caregiver's knowledge of online activity	"How much do your caregivers know about what you do online?"	"Almost everything" (1)<br>"Quite a lot" (2)<br>"Just a little" (3)<br>"Almost nothing" (4)	Child participant	9
	"Have you ever done anything online your caregivers don't know about?"	"Yes" (1)<br>"No" (0)	Child participant	9
	"Have you lied to your caregivers about what you do online?"	"Yes" (1)<br>"No" (0)	Child participant	9
Conduct problems	Strengths and difficulties questionnaire: Child___<br>1. "often has temper tantrums or hot tempers"<br>2. "is generally obedient"<br>3. "often fights with other children"<br>4. "often lies or cheats"<br>5. "steals from home, school or elsewhere"	"Not true" (0)<br>"Somewhat true" (1)<br>"Certainly true" (2)<br>Scoring reversed for generally obedient<br>Total score - /10<br>A higher score indicative of poorer conduct	Main carer	8
Law-breaking behaviours	"Have you ever stolen from a shop?"	"Yes" (1)<br>"No" (2)	Child participant	9
	"Have you ever stolen money?"	"Yes" (1)	Child participant	9

		“No” (2)		
	“Have you ever carried a knife?”	“Yes” (1)<br>“No” (2)	Child participant	9
	“Have you ever damaged a property?”	“Yes” (1)<br>“No” (2)	Child participant	9
	“Have you ever broken into a place?”	“Yes” (1)<br>“No” (2)	Child participant	9
	“Have you ever graffitied?”	“Yes” (1)<br>“No” (2)	Child participant	9
Smoking	“Have you ever tried a cigarette?”	“Yes” (1)<br>“No” (2)	Child participant	9
	“How often do you smoke?”	“Everyday” (1)<br>“At least once a week” (2)<br>“Less than once a week” (3)<br>“I do not smoke now” (4)	Child participant	9
	“How old were you when you first smoked a cigarette?”	“Age 8 or under” (1)<br>“Age 9 or 10” (2)<br>“Age 11 and over” (3)<br>“I have never smoked a whole cigarette” (4)	Child participant	9
	“Have you tried e-cigarettes or vaping?”	“Yes” (1)<br>“No” (2)	Child participant	9
Alcohol consumption	“Have you ever had an alcoholic drink?”	“Yes” (1)<br>“No” (2)	Child participant	9
	“How old were you when you had your first alcoholic drink?”	“Age 8 or under” (1)<br>“Age 9 or 10”(2)<br>“Age 11 and over” (3)	Child participant	9
	“How often have you drank alcohol in the last 30 days?”	“Never” (1)<br>“1-2 days” (2)<br>“3-5 days” (3)<br>“6-9 days” (4)<br>“10-19 days” (5)<br>“20-30 days” (6)	Child participant	9
	“Have you ever been drunk?”	“Yes” (1)<br>“No” (2)	Child participant	9

Supplementary material 2

Table S1.1, Differences in mean and confidence intervals of BMIp at 12 and 14-years (sweep 9 and 10), sleep duration at 14-years (sweep 10) by participation in team and individual sports at 12-years (sweep 9)

	Participation in team sports (S9) (no compared with yes)				Participation in individual sports (S9) (no compared with yes)			
	N	Mean difference	CI (95%) of the difference		N	Mean difference	CI (95%) of the difference	
			Lower	Upper			Lower	Upper
**BMIp (S9)**	3210	0.03	0.01	0.05	3210	0.03	0.01	0.05
**BMIp (S10)**	2133	0.02	-0.01	0.04	2133	0.02	-0.01	0.04
**Sleep duration (S10) (hours)**	2641	-0.33	-0.41	-0.24	2641	-0.16	-0.25	-0.08

Key: BMIp – body mass index percentile; CI – confidence interval; N – number; S – sweep

Table S1.2, Pearson correlation of the unadjusted longitudinal relationship between physical activity at 10-years (sweep 8), sleep duration at 14-years (sweep 10) and obesity at 12 and 14-years (sweeps 9 and 10)

	1	2	3	4
**1. Sleep duration (14yrs)**	--			
**2. BMIp (12yrs)**	-0.05	--		
**3. BMIp (14yrs)**	-0.05	0.85	--	
**4. Physical activity (hours per day) (10yrs)**	0.13	-0.04	-0.02	--

Table S2.1. Mean and confidence interval differences in BMIp at 12 and 14-years (sweeps 9 and 10), sleep duration at 14-years and screen time behaviours at 10 and 12-years (sweeps 8 and 9)

a. TV in the bedroom at 10-years, BMIp at 12 and 14-eyars and sleep duration at 14-years

	**TV in the bedroom (S8) (no compared with yes)**			
	**N**	**Mean difference**	**CI of the difference**	
			**Lower**	**Upper**
**BMIp (S9)**	2646	-0.06	-0.09	-0.04
**BMIp (S10)**	2034	-0.05	-0.07	-0.02
**Sleep duration (S10) (hours)**	2410	0.19	0.10	0.28

b. Computer in the bedroom at 10-years, BMIp at 12 and 14-eyars and sleep duration at 14-years

	**Computer in the bedroom (S8) (no compared with yes)**			
	**N**	**Mean difference**	**CI of the difference**	
			**Lower**	**Upper**
**BMIp (S9)**	2646	-0.05	-0.07	-0.03
**BMIp (S10)**	2034	-0.04	-0.06	-0.01
**Sleep duration (S10) (hours)**	2410	0.20	-0.11	0.28

c. Games console in the bedroom at 10-years, BMIp at 12 and 14-eyars and sleep duration at 14-years

	**Games console in the bedroom (S8) (no compared with yes)**			
	**N**	**Mean difference**	**CI of the difference**	
			**Lower**	**Upper**
**BMIp (S9)**	2646	-0.03	-0.06	-0.01
**BMIp (S10)**	2034	-0.03	-0.05	0.00
**Sleep duration (S10) (hours)**	2410	0.05	-0.04	0.13

d. Internet access in the bedroom at 10-years, BMIp at 12 and 14-eyars and sleep duration at 14-years

	Internet access in the bedroom (S8) (no compared with yes)			
	N	Mean difference	CI of the difference	
			Lower	Upper
**BMIp (S9)**	2646	-0.04	-0.07	-0.02
**BMIp (S10)**	2034	-0.03	-0.06	-0.01
**Sleep duration (S10) (hours)**	2410	0.21	0.12	0.29

e. Mobile phone in the bedroom at 10-years, BMIp at 12 and 14-eyars and sleep duration at 14-years

	Mobile phone in the bedroom (S8) (no compared with yes)			
	N	Mean difference	CI of the difference	
			Lower	Upper
**BMIp (S9)**	2646	-0.05	-0.07	-0.02
**BMIp (S10)**	2034	-0.04	-0.07	-0.01
**Sleep duration (S10) (hours)**	2410	0.22	0.13	0.31

f. Caregivers' knowledge of online activity at 12-years, BMIp at 12 and 14-eyars and sleep duration at 14-years

	Caregiver doesn't know of online activities (S9) (no compared with yes)			
	N	Mean difference	CI of the difference	
			Lower	Upper
**BMIp (S9)**	2991	0.04	0.00	0.08
**BMIp (S10)**	1971	0.03	-0.02	0.07
**Sleep duration (S10) (hours)**	2443	-0.20	-0.36	-0.03

g. Child lied to parents about online activity at 12-years, BMIp at 12 and 14-eyars and sleep duration at 14-years

	Lied to parents about online activities (S9) (no compared with yes)			
	**N**	**Mean difference**	**CI of the difference**	
			**Lower**	**Upper**
**BMIp (S9)**	2991	0.02	-0.02	0.06
**BMIp (S10)**	1971	0.02	-0.03	0.07
**Sleep duration (S10) (hours)**	2443	-0.35	-0.52	-0.18

Key: BMIp – body mass index percentile; CI – confidence interval; N – number; S - sweep

Table S2.2. Pearson correlation of the unadjusted longitudinal relationship between screen time and sleep and obesity

	**1**	**2**	**3**	**4**	**5**	**6**	**7**	**8**	**9**
**1. Sleep duration (14yrs)**	--								
**2. BMIp (12yrs)**	-0.05	--							
**3. BMIp (14yrs)**	-0.05	0.85	--						
**4. TV days (10yrs)**	0.00	0.05	0.06	--					
**5. TV WD (10yrs)**	-0.08	0.06	0.03	0.22	--				
**6. TV (hours) WE (10yrs)**	-0.07	0.03	0.03	0.18	0.27	--			
**7. Electronics (days) (10yrs)**	-0.03	0.04	0.02	0.10	0.05	0.02	--		
**8. Electronics (hours) WD (10yrs)**	-0.09	0.10	0.07	-0.03	0.16	0.02	0.26	--	
**9. Electronics (hours) WE (10yrs)**	-0.08	0.04	0.02	-0.01	0.07	0.17	0.19	0.34	--

Key: BMIp - body mass index percentile; hrs – hours; TV – television; WD – weekday; WE – weekend; yrs – years

Blue – Positive association (for example, as electronic use on weekdays increased, BMIp increased)

Yellow – Negative association (for example, as electronic use on weekdays increased, sleep duration decreased)

Table S3.1. Mean and standard deviations of BMIp at 12 and 14-years (sweep 9 and 10) and sleep duration at 14-years (sweep 10) by diet variable responses at 10-years (sweep 8)

a. Breakfast consumption at 10-years, BMIp at 12 and 14-years and sleep duration at 14-years

		Breakfast consumption (S8)			
		Never	Occasionally	Quite often	Always
BMIp (S9)	N	42	166	184	2254
	Mean	0.7	0.7	0.7	0.6
	SD	0.3	0.3	0.3	0.3
BMIp (S10)	N	35	111	139	1749
	Mean	0.7	0.7	0.7	0.6
	SD	0.2	0.3	0.3	0.3
Sleep Duration (S10)	N	38	122	165	2079
	Mean	8.7	8.6	8.5	8.9
	SD	1.8	1.9	2	1.7

b. Electronics during meals at 10-years, BMIp at 12 and 14-years and sleep duration at 14-years

		Electronics during meals (S8)			
		Never	Occasionally	Quite often	Always
BMIp (S9)	N	1091	978	353	219
	Mean	0.6	0.7	0.7	0.7
	SD	0.3	0.3	0.3	0.3
BMIp (S10)	N	830	759	272	169
	Mean	0.6	0.7	0.6	0.7
	SD	0.3	0.3	0.3	0.3
Sleep Duration (S10)	N	987	905	321	187
	Mean	8.9	8.8	8.9	8.7
	SD	1.7	1.7	1.7	2.1

c. Eat whatever at 10-years, BMIp at 12 and 14-years and sleep duration at 14-years

		Eat whatever they want (S8)			
		Never	Occasionally	Quite often	Always

BMIp (S9)	N	647	1657	266	75
	Mean	0.7	0.7	0.7	0.7
	SD	0.3	0.3	0.3	0.3
BMIp (S10)	N	494	1276	207	57
	Mean	0.6	0.6	0.7	0.6
	SD	0.3	0.3	0.3	0.3
Sleep Duration (S10)	N	591	1508	240	65
	Mean	8.8	8.8	8.7	9
	SD	1.7	1.7	1.9	2.1

d. Eat whenever at 10-years, BMIp at 12 and 14-years and sleep duration at 14-years

		Eat whenever they want (S8)			
		Never	Occasionally	Quite often	Always
BMIp (S9)	N	929	1465	203	48
	Mean	0.7	0.7	0.6	0.7
	SD	0.3	0.3	0.3	0.3
BMIp (S10)	N	724	1122	152	35
	Mean	0.7	0.6	0.6	0.6
	SD	0.3	0.3	0.3	0.3
Sleep Duration (S10)	N	848	1329	186	40
	Mean	8.9	8.8	8.5	8.5
	SD	1.7	1.7	1.9	2.3

e. Chocolate and sweets consumption at 10-years, BMIp at 12 and 14-years and sleep duration at 14-years

		Chocolate and sweets consumption (S8)				
		More than once a day	Once a day	5-6 days a week	2-3 days a week	Once a week
BMIp (S9)	N	264	975	306	783	234
	Mean	0.6	0.7	0.7	0.7	0.6
	SD	0.3	0.3	0.3	0.3	0.3
BMIp (S10)	N	204	752	227	598	179
	Mean	0.6	0.7	0.6	0.6	0.6
	SD	0.3	0.3	0.3	0.3	0.3
	N	236	891	275	706	221

Sleep Duration SN (S10)	Mean	7.8	7.9	8	7.9	8.1
	SD	1.1	1.1	1.1	1.1	1.1

f. Crisps consumption at 10-years, BMIp at 12 and 14-years and sleep duration at 14-years

		**Crisps consumption (S8)**				
		**More than once a day**	**Once a day**	**5-6 days a week**	**2-3 days a week**	**Once a week**
**BMIp (S9)**	**N**	82	753	326	838	367
	**Mean**	0.7	0.7	0.6	0.7	0.6
	**SD**	0.3	0.3	0.3	0.3	0.3
**BMIp (S10)**	**N**	64	572	253	649	284
	**Mean**	0.7	0.7	0.6	0.6	0.6
	**SD**	0.3	0.3	0.3	0.3	0.3
**Sleep Duration SN (S10)**	**N**	69	674	297	777	344
	**Mean**	7.8	7.9	7.8	8	7.9
	**SD**	1	1.1	1.1	1.1	1.1

g. *SSB consumption at 10-years, BMIp at 12 and 14-years and sleep duration at 14-years*

		**SSB consumption (S8)**				
		**More than once a day**	**Once a day**	**5-6 days a week**	**2-3 days a week**	**Once a week**
**BMIp (S9)**	**N**	430	355	92	274	334
	**Mean**	0.65	0.64	0.68	0.7	0.64
	**SD**	0.3	0.31	0.29	0.29	0.3
**BMIp (S10)**	**N**	318	273	71	228	253
	**Mean**	0.64	0.62	0.62	0.68	0.64
	**SD**	0.3	0.31	0.3	0.28	0.27
**Sleep Duration SN (S10)**	**N**	375	326	86	272	291
	**Mean**	7.81	7.94	7.77	7.97	8.04
	**SD**	1.23	1.11	1.05	1.05	1.01

Key: BMIp – body mass index percentile; N – number; S – sweep; SSB – sugar-sweetened beverage. Pink – higher BMIp; shorter sleep duration; green – lower BMIp; longer sleep duration.

Table S3.2. Pearson correlations of the longitudinal relationship between diet choices at 10-years (sweep 8) and sleep duration at 14-years (sweep 10) and obesity at 12 and 14-years (sweeps 9 and 10)

	**1**	**2**	**3**	**4**	**5**
**1. Sleep duration (14yrs)**	--				
**2. BMIp (12yrs)**	-0.05	--			
**3. BMIp (14yrs)**	-0.05	0.85	--		
**4. Fruit consumption (10yrs)**	0.05	-0.01	0.01	--	
**5. Vegetable consumption (10yrs)**	0.06	-0.02	-0.03	0.23	--

Key: BMIp – body mass index percentile; yrs – years

Blue – Positive association (for example, as the vegetable consumption increased, sleep duration increased)

Yellow – Negative association (for example, as vegetable consumption increased, bedtime became earlier)

Table S4.1. Mean and confidence interval differences in BMIp at 12 and 14-years, sleep duration at 14-years and risk-taking at 12 and 14-years

a. Smoking at 12-years, BMIp at 12 and 14-years and sleep duration at 14-years

	Tried a cigarette (S9) (yes compared with no)			
	N	Mean difference	CI of the difference	
			Lower	Upper
**BMIp (S9)**	3178	0.13	0.07	0.18
**BMIp (S10)**	2095	0.10	0.02	0.17
**Sleep duration (S10) (hours)**	2593	-0.54	-0.79	0.29

b. Vaping at 12-years, BMIp at 12 and 14-years and sleep duration at 14-years

	Tried vaping (S9) (yes compared with no)			
	N	Mean difference	CI of the difference	
			Lower	Upper
**BMIp (S9)**	3178	0.09	0.05	0.14
**BMIp (S10)**	2095	0.07	0.01	0.12
**Sleep duration (S10) (hours)**	2593	-0.26	-0.46	-0.07

c. Drinking alcohol at 12-years, BMIp at 12 and 14-years and sleep duration at 14-years

	Tried alcohol (S9) (yes compared with no)			
	N	Mean difference	CI of the difference	
			Lower	Upper
**BMIp (S9)**	3178	0.06	0.04	0.09
**BMIp (S10)**	2095	0.07	0.04	0.11
**Sleep duration (S10) (hours)**	2593	-0.24	-0.35	-0.13

d. Stolen from a shop at 12-years, BMIp at 12 and 14-years and sleep duration at 14-years

	Stolen from a shop (S9) (yes compared with no)			
	N	Mean difference	CI of the difference	
			Lower	Upper
**BMIp (S9)**	3178	0.04	-0.01	0.08

**BMIp (S10)**	2095	0.04	-0.02	0.09
**Sleep duration (S10) (hours)**	2593	-0.44	-0.63	-0.25

e. Stolen money at 12-years, BMIp at 12 and 14-years and sleep duration at 14-years

	**Stolen money (S9) (yes compared with no)**			
	**N**	**Mean difference**	**CI of the difference**	
			**Lower**	**Upper**
**BMIp (S9)**	3178	0.03	<-.001	0.07
**BMIp (S10)**	2095	0.04	<.001	0.08
**Sleep duration (S10) (hours)**	2593	-0.22	-0.36	-0.07

f. Carried a weapon at 12-years, BMIp at 12 and 14-years and sleep duration at 14-years

	**Carried a weapon (S9) (yes compared with no)**			
	**N**	**Mean difference**	**CI of the difference**	
			**Lower**	**Upper**
**BMIp (S9)**	3178	0.01	-0.08	0.09
**BMIp (S10)**	2095	-0.02	-0.12	0.09
**Sleep duration (S10) (hours)**	2593	-0.12	-0.49	0.24

Key: BMIp – body mass index percentile; CI – confidence interval; N – number; S – sweep.

Table S4.2. The longitudinal relationship between risk-taking behaviours and sleep and obesity: Pearson correlation

	**1**	**2**	**3**	**4**	**5**
**1. Sleep duration (14yrs)**	--				
**2. BMIp (12yrs)**	-0.05	--			
**3. BMIp (14yrs)**	-0.05	0.85	--		
**4. Conduct (10yrs)**	-0.13	0.05	0.05	--	
**5. Conduct (12yrs)**	-0.10	0.06	0.05	0.64	--

Key: BMIp – Body mass index percentile; yrs – years

Blue – Positive association (for example, as conduct score increased, BMIp increased)

Yellow – Negative association (for example, as conduct score increased, sleep duration decreased)
